# Effects of body condition on the insulin resistance, lipid metabolism and oxidative stress of lactating dairy cows

**DOI:** 10.1186/s12944-020-01233-7

**Published:** 2020-03-30

**Authors:** Jiajin Wu, Jianxin Liu, Diming Wang

**Affiliations:** Institute of Dairy Science, MoE Key Laboratory of Molecular Animal Nutrition, College of Animal Sciences, Zhejiang University, Hangzhou, 310058 People’s Republic of China

**Keywords:** Body condition score, Insulin resistance, Lipolysis, Oxidative stress, Hematological parameters

## Abstract

**Background:**

Overconditioned dairy cows are prone to greater insulin resistance in transition to successfully adapt to negative energy balance. The associations among body condition score (BCS), insulin resistance, lipid metabolism and oxidative stress in cows during late lactation with positive energy balance remain to be elucidated.

**Methods:**

The objectives of this study were to investigate insulin sensitivity and oxidative status in late lactating dairy cows with different BCS but similar milk production, parity and days in milk. Forty-two multiparous Holstein cows were fed the same diet under the same management and divided into three groups based on BCS: low BCS (LBCS; BCS ≤ 2.75; *n* = 12), medium BCS (MBCS; 3.0 ≤ BCS ≤ 3.5; *n* = 15) or high BCS (HBCS; BCS ≥ 3.75; n = 15). Blood samples used for analysis of biochemical and hematological parameters were collected from the coccygeal vein at the end of experiment.

**Results:**

The concentrations of insulin and nonesterified fatty acid were higher and the revised quantitative insulin sensitivity check index (RQUICKI) was lower in HBCS cows than in LBCS and MBCS cows. These results suggest that insulin resistance exacerbates lipolysis in HBCS cows. Serum concentrations of very low-density lipoprotein, apolipoprotein A-I, and apolipoprotein B were lower in HBCS cows than in LBCS or MBCS cows. Although LBCS and MBCS cows had higher reactive oxygen species levels than did HBCS cows, the malondialdehyde concentration was not different among cows with different BCS. Ceruloplasmin activity was higher in MBCS and HBCS cows than in LBCS cows, but superoxide dismutase, glutathione peroxidase, and paraoxonase activities were not different among cows with different BCS. Despite the higher percentage of granulocytes in MBCS cows than in HBCS cows, no differences were found in leukocyte counts, red blood cell profiles and platelet profiles among the cows in the three groups.

**Conclusions:**

Results of this study showed that compared with MBCS and LBCS cows, HBCS cows at late lactation stage may have accumulated more hepatic triacylglycerol and lower antioxidant potential due to greater insulin resistance.

## Background

An appropriate body condition score (BCS) plays an important role in maintaining the health status of dairy cows [1]. The recommended acceptable BCS of dairy cows ranges from 3.0 to 3.5 on a 5-point scale to ensure that health, reproduction and animal welfare are not compromised [2]. A previous study showed that cows with low BCS (LBCS) are more susceptible to metabolic diseases and claw diseases [3]. In contrast, cows with high BCS (HBCS) suffer from many metabolic disorders, such as fatty liver, oxidative stress and ketosis [4]. Cows with HBCS are more prone to develop insulin resistance than are cows with medium BCS (MBCS) and LBCS. A transient state of insulin resistance in transition and early lactating dairy cows is generally considered a homeorhetic adaptation, which guarantees glucose supply to the gravid uterus and to the lactating mammary gland by limiting glucose use by peripheral insulin-responsive tissues (such as skeletal muscles and adipose tissue) [5, 6]. However, insulin resistance can exacerbate the lipolysis of adipose tissue and the accumulation of nonesterified fatty acid (NEFA) in turn leads to greater insulin resistance, which is associated with health problems such as fatty liver and oxidative stress [6, 7].

Compared with healthy cows, cows with fatty liver have lower concentrations of apolipoprotein A-I (apo A-I) [8, 9]. Apo A-I and paraoxonase (PON) are chain-breaking antioxidants that can remove reactive oxygen species (ROS) [10]. A recent study found lower PON activity in HBCS cows than in optimal BCS cows after parturition; however, no difference was found in apoA-I concentration, partly due to lack of difference in NEFA concentration between the two groups of cows [11]. NEFA can modify the intracellular production of ROS [12], and higher concentrations of NEFA in HBCS cows after calving were reported to diminish the generation of ROS in neutrophils [13]. However, a previous study showed that low and moderate concentrations of NEFA (0.0625 ~ 0.5 mmol/l) induced a decrease in the generation of ROS in neutrophils, whereas high concentrations (1 ~ 2 mmol/l) did not cause a difference from the control in vitro [14]. HBCS not only alters the function of bovine peripheral blood immune cells but is also associated with their counts and other hematological parameters [13, 15]. Although previous studies have suggested that cows with HBCS have an increased NEFA concentration [7, 16], it was recently reported that the serum NEFA of HBCS cows was not higher than that of optimal BCS cows partly due to lower milk yield, which could indicate a lower negative energy balance status [17]. Additionally, cows have HBCS when they are fed total mixed rations at the late lactation stage [18], and some cows with high milk production have LBCS at the end of the lactation cycle [1], suggesting that maintaining late lactating cows under an appropriate BCS is important for the health status of cows in the close-up stage.

Considering that the effects of BCS on the glucolipid metabolism and oxidative stress of late lactating dairy cows remain unclear, we hypothesized that a high degree of insulin resistance occurred in cows with HBCS at the late lactation stage and that their high serum NEFA concentrations affected lipoprotein metabolism, oxidative status, and hematological parameters. Therefore, the objective of this study was to determine insulin resistance, oxidative status and hematological parameters in the blood of late lactating cows with different BCS but similar milk production, parity, and days in milk.

## Methods

The experimental procedures were performed in accordance with a protocol approved by the Animal Care Committee at Zhejiang University (Hangzhou, China). Forty-two multiparous Holstein cows with similar milk production, parity and days in milk were housed in an open barn and fed the same diet. BCS was recorded three times within a month, and the cows were retrospectively divided into three groups based on a 5-point scale BCS [19]: LBCS (BCS ≤ 2.75; *n* = 12), MBCS (3.0 ≤ BCS ≤ 3.5; *n* = 15) or HBCS (BCS ≥ 3.75; n = 15). Information regarding the experimental cows is shown in Table 1; the milk yield and milk composition were recorded for three consecutive days, and the 305-day milk production of last lactation were also similar among the three groups (data not shown). The animals were fed three times daily at 0630, 1400, and 2000 and had free access to drinking water.

**Table 1:** Basic information on dairy cows with different body condition score (BCS)

Item	BCS^1^			SEM	*P*-value
	Low	Medium	High		
No. of cows, head	12	15	15		
Parity	2.8	2.3	2.3	0.24	0.23
Days in milk	220	236	233	5.77	0.16
BCS	2.68^c^ (2.50–2.75)	3.22^b^ (3.00–3.50)	3.88^a^ (3.75–4.25)	0.04	< 0.01
Milk yield, kg/d	31.7	32.0	30.2	0.83	0.23
*Milk composition, %*					
Fat	3.91	3.85	3.86	0.26	0.99
Protein	3.21^b^	3.50^a^	3.38^ab^	0.06	0.01
Lactose	5.07	5.14	5.18	0.05	0.32

^a-c^ Means within same row with different superscripts differ (*P* < 0.05)

^1^ Low: BCS ≤ 2.75 (*n* = 12); Medium: 3.0 ≤ BCS ≤ 3.5 (*n* = 15); BCS ≥ 3.75 (*n* = 15)

Blood samples were collected from the coccygeal vein using serum tubes and EDTA tubes 3 h after the morning feeding at the end of the experiment. The blood samples in serum tubes were placed immediately on ice, and maintained at 4 °C for at least 2 h to allow for coagulation. The samples were then centrifuged at 3000×*g* at 4 °C for 30 min to collect the serum, which was then frozen at − 20 °C. The serum samples were analyzed with an Auto Analyzer 7020 instrument (Hitachi High-Technologies Corporation, Tokyo, Japan) using commercially obtained colorimetric detection kits (Nanjing Jiancheng Bioengineering Institute., Nanjing, China) for glucose, NEFA, β-hydroxybutyric acid (BHBA), triacylglycerol, cholesterol, high-density lipoprotein cholesterol (HDL-C), gamma-glutamyl transpeptidase, aspartate aminotransferase, alkaline phosphatase, albumin, bilirubin, ceruloplasmin, and malondialdehyde (MDA). Commercial ELISA kits (Nanjing Jiancheng Bioengineering Institute., Nanjing, China) were used to analyze serum insulin, glucagon, very low-density lipoprotein (VLDL), apo A-I, apolipoprotein B (apo B), superoxide dismutase (SOD), glutathione peroxidase (GSH-Px), PON, myeloperoxidase, and ROS. To analyze insulin sensitivity, the revised quantitative insulin sensitivity check index (RQUICKI) was calculated using the following equation [20]: RQUICKI = 1/[log glucose (mg/dL) + log insulin (μIU/mL) + log NEFA (mmol/L)].

The EDTA-treated full blood samples were analyzed using a Mindray BC-2600 automated hematologic analyzer (Mindray Medical International Co., Ltd., Shenzhen, China) to determine the counts and percentage of granulocytes, monocytes, and lymphocytes and the profiles of red blood cells and blood platelets.

All data analysis was performed with the PROC MIXED procedure of SAS software (version 9.0, SAS Institute Inc., Cary, NC). BCS was considered to be a fixed variable and each cow within the BCS group was treated as a random variable. An autoregressive covariate structure was used. To estimate the effects of the BCS group on the experimental variables, the least squares means were separated using the PDIFF statement of SAS. Data were considered significantly different at *P* <  0.05, and trends were defined at 0.05 ≤ *P* ≤ 0.10.

## Results

To determine the differences of insulin resistance among cows with different BCS, we measured metabolites related with insulin metabolism. The results are presented in Table 2. The serum insulin concentration in HBCS cows was higher than that in LBCS (*P* <  0.01) and MBCS cows (*P* = 0.01), with no difference between MBCS and LBCS cows (*P* > 0.10). Moreover, the serum NEFA concentration in HBCS cows was higher than that in MBCS cows (*P* <  0.05) and tended to be higher than that in LBCS cows (*P* = 0.06), with no difference between MBCS and LBCS cows (*P* > 0.10). The RQUICKI value was higher in LBCS (*P* <  0.05) and MBCS cows (*P* <  0.05) than in HBCS cows, with no difference between LBCS and MBCS cows (*P* > 0.10). Serum concentrations of glucose, glucagon (*P* = 0.72) and BHBA (*P* = 0.38) were not different among cows with different BCS (Table 2).

**Table 2:** Serum energy metabolism, lipid and lipoprotein parameters in cows with different body condition score (BCS)

Item	BCS^1^			SEM	*P*-value
	Low	Medium	High		
*Energy metabolism parameters*^*2*^					
Glucose, mmol/L	3.20	3.22	3.27	0.07	0.72
Insulin, mIU/L	19.7^b^	20.5^b^	24.1^a^	1.01	< 0.01
Glucagon, pg/mL	103	111	104	5.9	0.58
NEFA, μmol/L	119^ab^	116^b^	134^a^	5.53	0.06
BHBA, mmol/L	0.56	0.62	0.57	0.03	0.38
RQUICKI	0.47^a^	0.47^a^	0.44^b^	0.01	< 0.01
*Lipid and lipoprotein parameters*^*3*^					
Triacylglycerol, μmol/L	32.2^b^	34.3^b^	50.1^a^	5.59	0.06
Cholesterol, mmol/L	6.57	6.46	6.45	0.20	0.90
VLDL, mmol/L	1.80^ab^	1.97^a^	1.53^b^	0.11	0.03
HDL-C, mmol/L	0.43	0.47	0.46	0.02	0.33
apo A-I, mg/L	294^a^	280^a^	242^b^	12.9	0.02
apo B, mg/L	174^a^	176^a^	155^b^	5.1	0.01

^a-b^ Means within same row with different superscripts differ (*P* < 0.05)

^1^Low: BCS ≤ 2.75 (*n* = 12); Medium: 3.0 ≤ BCS ≤ 3.5 (*n* = 15); BCS ≥ 3.75 (*n* = 15)

^2^*NEFA* non-esterified fatty acid; *BHBA* β-hydroxybutyric acid; *RQUICKI* revised quantitative insulin sensitivity check index, calculated using the following equation (Leiva et al., 2014): *RQUICKI* 1/ [log glucose (mg/dL) + log insulin (μIU/mL) + log NEFA (mmol/L)]

^3^*VLDL* very low-density lipoprotein; *HDL-C* high-density lipoprotein cholesterol; *apo A-I* apolipoprotein A-I; *apo B* apolipoprotein B

To determine whether insulin resistance difference between cows with different BCS led to their variation in liver lipid metabolism, parameters related with lipid and lipoprotein were further evaluated. The serum triacylglycerol concentration of HBCS cows was higher than that of LBCS and MBCS cows (*P* <  0.05), with no difference between LBCS and MBCS cows (*P* > 0.10). The serum VLDL concentration was lower in HBCS cows than in MBCS cows (*P* < 0.01) and LBCS cows (*P* = 0.10), while serum apo A-I and apo B concentrations were higher in LBCS (*P* < 0.05) and MBCS cows (*P* < 0.05) compared to HBCS cows, with no difference (*P* > 0.10) between LBCS and MBCS cows. Serum cholesterol and HDL-C concentrations were not different (*P* > 0.10) among the cows in the three groups.

**Fig. 1:**
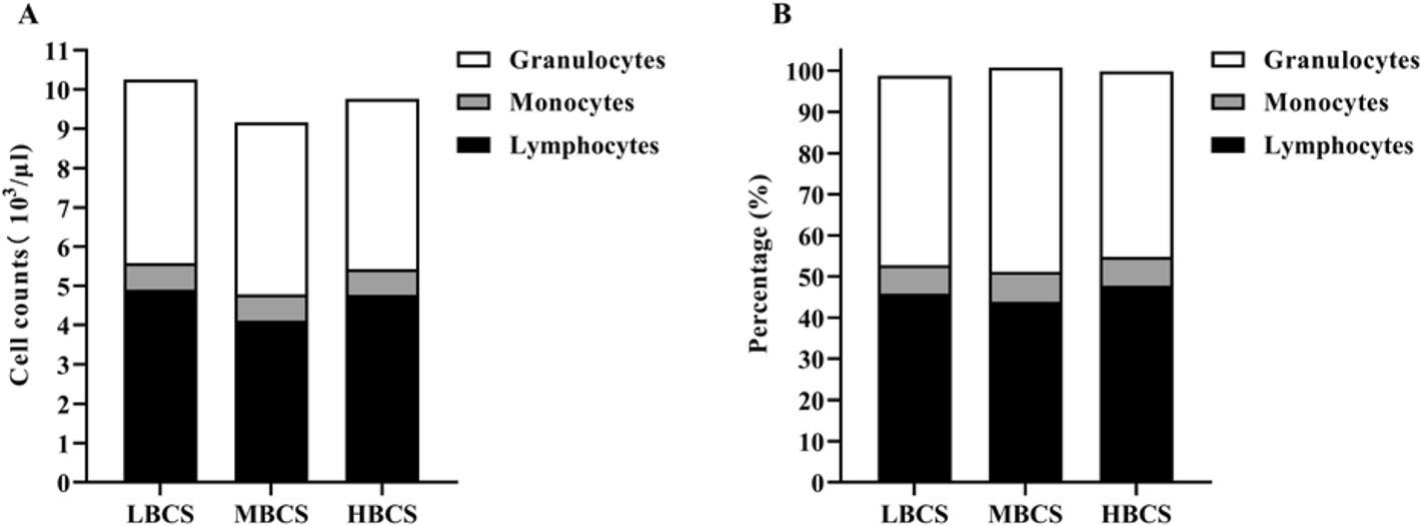
Effects of body condition score (BCS) on peripheral blood immune cells. **A.** the total counts of granulocytes, monocytes, and lymphocytes; **B.** the percentage of granulocytes, monocytes, and lymphocytes. LBCS, BCS ≤ 2.75 (*n* = 12); MBCS, 3.0 ≤ BCS ≤ 3.5 (*n* = 15); HBCS, BCS ≥ 3.75 (*n* = 15)

We further determined the liver function and oxidative stress related index in the blood of cows with different BCS (Table 3). The concentration of albumin was lower (*P* < 0.05) in LBCS cows compared to HBCS cows, with no difference (*P* > 0.10) between MBCS cows and LBCS or HBCS cows. Serum concentrations of gamma-glutamyl transpeptidase, aspartate aminotransferase, alkaline phosphatase, and bilirubin were not affected (*P* > 0.10) by BCS. Serum concentrations of SOD (*P* = 0.78), GSH-Px (*P* = 0.64), PON (*P* = 0.59), myeloperoxidase (*P* = 0.47), and MDA (*P* = 0.41) were not different among cows with LBCS, MBCS and HBCS (*P* > 0.10). The activity of serum ceruloplasmin was lower in LBCS cows than in MBCS (*P* < 0.01) and HBCS cows (*P* = 0.07) but not different (*P* > 0.10) between MBCS and HBCS cows. The serum ROS concentration of MBCS (*P* < 0.01) and LBCS cows (*P* = 0.09) was higher than in that of HBCS cows, with no difference between LBCS and MBCS cows (*P* > 0.10). Finally, we investigated if the altered redox status would be related to hematological profiles (Table 4). The total counts of granulocytes, monocytes, and lymphocytes were not affected by BCS (Fig. 1a). The percentage of granulocytes tended to be higher (*P* = 0.07) in MBCS cows than in HBCS cows, with no difference between LBCS cows and MBCS or HBCS cows. BCS had no effect on the percentage of monocytes or lymphocytes (Fig. 1b). The red blood cell profile, including red blood cell count, hemoglobin, hematocrit, mean corpuscular volume, and mean corpuscular hemoglobin, were not different (*P* > 0.10) across cows in the three groups (Table 4). Platelet count, mean platelet volume, platelet distribution width, and thrombocytocrit were also not affected (*P* > 0.10) by BCS.

**Table 3:** Serum biomarkers of liver function and oxidative stress in dairy cows with different body condition score (BCS)

Item^2^	BCS^1^			SEM	*P*-value
	Low	Medium	High		
GGT, U/L	26.5	30.1	30.4	1.59	0.19
AST, U/L	67.6	62.9	68.0	2.92	0.39
ALP, U/L	52.3	48.5	51.2	2.70	0.61
Albumin, g/L	30.9^b^	31.5^ab^	32.3^a^	0.35	0.03
Bilirubin, μmol/L	1.89	1.99	1.86	0.06	0.21
SOD, U/mL	62.2	65.0	62.9	2.91	0.78
GSH-Px, U/mL	163	159	169	8.2	0.64
PON, IU/L	841	812	875	48.3	0.59
Ceruloplasmin, U/L	18.1^b^	27.1^a^	23.8^ab^	2.11	0.02
Myeloperoxidase, U/L	9.40	9.62	8.94	0.41	0.47
ROS, IU/mL	134^ab^	140^a^	127^b^	3.1	< 0.01
MDA, nmol/mL	7.40	6.60	7.13	0.42	0.41

^a-b^Means within same row with different superscripts differ (*P* < 0.05)

^1^Low: BCS ≤ 2.75 (*n* = 12); Medium: 3.0 ≤ BCS ≤ 3.5 (*n* = 15); BCS ≥ 3.75 (*n* = 15)

^2^*GGT* gamma-glutamyltranspeptidae; *AST* aspartate aminotransferase; *ALP* alkaline phosphatase; *SOD* superoxide dismutase; *GSH-Px* glutathione peroxidase; *PON* paraoxonase; *ROS* reactive oxygen species; *MDA* malondiadehyde

**Table 4:** Hematological variables in cows with different body condition score (BCS)

Item	BCS^1^			SEM	*P*-value
	Low	Medium	High		
Red blood cells, 10^3^ /μL	6.30	6.36	6.44	0.14	0.75
Haemoglobin, g/L	91.8	91.4	95.2	1.50	0.14
Haematocrit, %	30.2	30.7	31.3	0.66	0.54
Mean corpuscular volume, fL	48.5	49.2	48.5	0.85	0.81
Mean corpuscular haemoglobin, pg	14.4	14.6	14.6	0.23	0.75
Platelet, 10^3^/μL	453	447	463	33.8	0.94
Mean platelet volume, fL	5.59	5.45	5.46	0.13	0.73
Platelet distribution width	16.6	16.4	16.5	0.17	0.60
Thrombocytocrit, %	0.24	0.24	0.26	0.02	0.69

^a-b^Means within same row with different superscripts differ (*P* < 0.05)

^1^Low: BCS ≤ 2.75 (n = 12); Medium: 3.0 ≤ BCS ≤ 3.5 (n = 15); BCS ≥ 3.75 (*n* = 15)

## Discussion

The higher insulin and lower RQUICKI of HBCS cows relative to LBCS and MBCS cows indicated that late lactating cows with HBCS are more prone to insulin resistance. This finding is consistent with a previous study conducted in early lactating cows [21]. Glucagon is also a primary regulator of glucose concentration. Glucagon concentration is relatively stable in late lactating cows, and milk production is positively associated with glucagon concentration in serum [22]. However, in the current study, no difference in serum glucagon was observed among the cows in the three groups, indicating that similar glucagon concentrations may be attributed to similar milk production among cows with LBCS, MBCS and HBCS. A previous study found no difference in BHBA concentration between cows with optimal and HBCS during the early lactating stage [11]. Similar results were observed in our study; BHBA concentrations were not affected by BCS, indicating similar NEFA oxidation in the liver of cows with LBCS, MBCS, and HBCS. In addition, the higher NEFA concentrations of HBCS cows compared with those of MBCS and LBCS cows in this study suggested a greater lipolysis status in cows with HBCS, consistent with a previous study conducted in transition cows [7]. The higher serum NEFA concentration in HBCS cows in our study may be attributed to the low insulin sensitivity that could increase NEFA flux [23]. These results suggested that compared with LBCS and MBCS cows, HBCS cows had greater insulin resistance.

The lipid and lipoprotein metabolism of lactating dairy cows can be disturbed by insulin resistance [6], especially when cows are in the HBCS range during the late pregnancy and early lactation stages [24]. Our results showed that during the late lactation stage, the serum triacylglycerol concentration was significantly higher in HBCS cows than in MBCS and LBCS cows. Low insulin sensitivity in HBCS cows might downregulate tissue lipoprotein lipase activity and VLDL-triacylglycerol utilization, which in turn appears to increase serum triacylglycerol [24, 25]. Leiva et al. (2018) approved that reduction of insulin resistance would lead to inhibition of lipolysis in HBCS-cows [20]. Moreover, the synthesis and secretion of apolipoproteins that limit VLDL assembly and secretion were lower in ruminants as compared to nonruminants [9]. A recent study found that compared to thin cows, HBCS cows had higher concentration of liver triacylglycerol [24]. In our study, apo A-I, apo B, and VLDL concentrations were lower in HBCS cow than in MBCS and LBCS cows, which is consistent with previous studies in which cows with fatty liver had significantly lower concentrations of apo A-I, apo B, and VLDL [9, 26]. Therefore, during the late lactation stage, cows with HBCS accumulated more hepatic triacylglycerol than did LBCS and MBCS cows, which is partly attributed to their greater insulin resistance.

The substantial generation of ROS accompanied by increased metabolic demands threatens cellular membrane integrity in mammalian tissues [27, 28]. Antioxidant defense systems in metabolic tissues, such as SOD, ceruloplasmin and chain-breaking antioxidants, including apo A-I and PON, can reduce ROS accumulation [11, 27]. Apo A-I not only plays a role in the synthesis and secretion of lipoprotein as discussed above, but also acts an antioxidant in dairy cows. Thus, the lower apo A-I concentrations in HBCS cows than in MBCS and LBCS cows indicated that HBCS cows may have lower antioxidant potential. In addition, apo A-I plays a role in PON stability on HDL particles [29]. However, no differences in PON activity were observed among the three groups of cows in this study, which is inconsistent with a previous study that reported lower PON activity in HBCS cows than in optimal BCS cows after parturition [11]. PON activity may be relatively stable during lactation, but it decreases around parturition due to metabolic disorders that frequently occur in this period [30].

Decreased antioxidant capacity and increased oxidant stress were observed in HBCS cows during the transition period and the mid-lactation stage [7, 31]. Although MBCS cows had higher levels of serum ROS, this increase did not result in greater lipid peroxidation in these cows in our study, as serum MDA concentration was not different among the cows in the three groups. This effect may be a result of higher serum ceruloplasmin concentration in cows with MBCS and HBCS compared with cows with LBCS. Ceruloplasmin, as an antioxidant, inhibits lipid peroxidation processes [32]. In addition, our results showed that bovine liver function was not affected by BCS. Although differences existed in the albumin concentrations among the three groups of cows in this study, their concentrations were still within the normal physiological range [33]. Thus, the lower antioxidant potential of HBCS cows may be due to greater insulin resistance-dependent lipoprotein metabolism.

The oxidative burst activity of neutrophils that defend against infectious diseases is also a source of ROS production [34]. A previous study showed that higher serum NEFA concentrations in HBCS cows decreased the generation of ROS in bovine neutrophils but did not affect the granulocyte (mostly neutrophil) counts and percentages or other hematological variables [13]. Although our results showed a higher NEFA and lower percentage of granulocytes in HBCS cows than in MBCS cows, the difference in ROS levels may not be due to differences in the generation of ROS by neutrophils in these groups because the activity of myeloperoxidase, an enzyme secreted by activated neutrophils and monocytes, was not different among the cows in the three groups in this study. A previous study also reported that no difference was observed in the ROS generation of neutrophils incubated with low and moderate NEFA (0.0625 ~ 0.5 mmol/l) in vitro [14]. Therefore, further studies are required to reveal how BCS affects the generation of ROS in dairy cows.

## Conclusions

In conclusion, compared to cows with MBCS or LBCS, cows with HBCS had more triacylglycerol accumulation in the liver due to their greater insulin resistance in the late lactation stage. Cows with HBCS may have lower antioxidant potential than cows with MBCS or LBCS. Our work highlighted that during late lactation, HBCS should be prevented in lactating cows to maintain a healthier status even though they were not in negative energy balance or severe oxidative stress.

## Data Availability

The datasets used or analyzed during the current study are available from the corresponding author on reasonable request.

## Abbreviations

ALP: Alkaline phosphatase
apo A-I: Apolipoprotein A-I
apo B: Apolipoprotein B
AST: Aspartate aminotransferase
BCS: Body condition score
BHBA: β-hydroxybutyric acid
DIM: Days in milk
GGT: Gamma-glutamyl transpeptidase
GSH-Px: Glutathione peroxidase
HDL-C: High-density lipoprotein cholesterol
MDA: Malondialdehyde
NEB: Negative energy balance
NEFA: Non-esterified fatty acid
PON: Paraoxonase
ROS: Reactive oxygen species
RQUICKI: Revised quantitative insulin sensitivity check index
SOD: Superoxide dismutase
VLDL: Very low-density lipoprotein
